# Supplementary material for: Loss of inner kinetochore genes is associated with the transition to an unconventional point centromere in budding yeast
Source: PeerJ. 2020 Sep 29;8:e10085. doi: 10.7717/peerj.10085 (PMC7531349; doi:10.7717/peerj.10085)
Supplement: Supplemental Information 3 — The multiple sequence alignment of open reading frame nucleotide sequences and amino acid sequences generated using MUSCLE, M-coffee, Clustal Omega and Guidance with PRANK are provided along with the commands used for the command line tools. Results of the selection tests from BUSTED, FEL, MEME and RELAX from the HYPHY package are provided as json files that can be visualized using the online hyphy vision website. [file peerj-08-10085-s003.zip › Supplementary_Material_S3/AA/M-Coffee_result_CSE4_AA.score_html]

T-COFFEE, Version\_11.00.d625267 (2016-01-11 15:25:41 - Revision d625267 - Build 507)  
Cedric Notredame   
CPU TIME:0 sec.  
SCORE=687  
\*  
 BAD AVG GOOD  
\*  
YKL049C        :  61  
Smik\_11.199    :  65  
Skud\_11.180    :  65  
Suva\_11.177    :  65  
CAGL0M13145g   :  62  
KAFR0I01590    :  67  
KNAG0H00580    :  49  
NCAS0H03050    :  44  
NDAI0C00590    :  46  
TBLA0G01890    :  49  
TPHA0N01420    :  55  
Kpol\_181.3     :  59  
ZYRO0F01144g   :  61  
TDEL0B06850    :  68  
KLLA0C12529g   :  68  
ABR083C        :  60  
Ecym\_2307      :  62  
SAKL0B11198g   :  67  
KLTH0D08602g   :  68  
Kwal\_26.8328   :  66  
cons           :  68  
  
YKL049C        MSSKQQWVSSAIQS-----------------DSSGRSLSN-------------------------V  
Smik\_11.199    MSSKQQWANSAIQS-----------------DSSGRSLSN-------------------------V  
Skud\_11.180    MSSKQQWANSAIQS-----------------DSSGRSLSN-------------------------V  
Suva\_11.177    MSSKQEWANSAIQS-----------------ESSGRSLSN-------------------------V  
CAGL0M13145g   MSTRQAVFERDVDE-----------------DRWPRSARGL-------------------------  
KAFR0I01590    MQS-QQWEQSN------------VNR----------------GLLE--------------------  
KNAG0H00580    M-------QKQQFD-----------------SSDWDLLPT-------------------------A  
NCAS0H03050    MSS-RKFVEQGHAQNSSSHLFSNILD------N-------DSGSLSNINRLTLDPDN---------  
NDAI0C00590    MQS-RKWIPSDRGNESTSHLFSSVNNSANNNYERARSIHNVNGLLPSIIAHRINEEDEDEEEDADF  
TBLA0G01890    ---------------MSQRTISNINR-----------------LND--------------------  
TPHA0N01420    -----------MET-----------------NGSNRTLTN-------------------------V  
Kpol\_181.3     M------LSSRIPN-----------------NSGNRALSN-------------------------I  
ZYRO0F01144g   METR----QLDGAV-----------------HNGSRLLSQ-------------------------L  
TDEL0B06850    MELKQLDHNASIHS-----------------DSGARTLSN-------------------------V  
KLLA0C12529g   M-------EQSIRSI-----------------DGSRSLSN-------------------------V  
ABR083C        M-------EQSMSS-----------------EQAERVAGR-------------------------V  
Ecym\_2307      M-------EQSIRN-----------------ELPSRNL---------------------------V  
SAKL0B11198g   MD-RQ-EWNASISS-----------------QTG-RSLSN-------------------------T  
KLTH0D08602g   M-------NASIAS-----------------QTGGRSLSN-------------------------T  
Kwal\_26.8328   M-------NASIAS-----------------QVGGRSLSN-------------------------T  
  
cons                                                                               
  
  
YKL049C        NRLA-----GDQQSINDRALSLLQRTRATKNLFPRR-E---ER-RRYESSKSDLDIET--------  
Smik\_11.199    NRLV-----GEQQSINDRALSLLQRTRARKNLFPRR-E---ER-RRYENPKDDVDFET--------  
Skud\_11.180    NRLV-----EDQQSINDRALSLLQRTRARKNLFPKR-E---ER-RRYEGPQDDMIFEE--------  
Suva\_11.177    NRLA-----GDQQSINDRALSLLQRTRARKNLFPRR-E---ER-RRYEGSQDDVEFEA--------  
CAGL0M13145g   AGVN-TVF-EGDSEINSKAMRLLEKTRHRRNLLNRRE----DR-RRYLGGVKAKAIES--------  
KAFR0I01590    ----------EQELINERANLLLQRTRERRNLLLQGTG--EHG-NLLLQGTT--DLLP--------  
KNAG0H00580    PL-T-----TDQEKINERARELLERNRRYGRLLKTGGDPLPER-RRYERDLDSTDIQDGDVVSNSG  
NCAS0H03050    ----TEDL-LQQEVINERALSLLQRTRERRNLLHRFED----KRRYYNQGQDDGDLES--------  
NDAI0C00590    DDLDADLR-LEQDDIDQKALLLLQRTRERKNLLNGQLPPEEDR-GRYYQNTMNDDIGS--------  
TBLA0G01890    ----------EQTLLNQRAAQFLQRNIQGRRLFQRY-K---ET-KRFENSNSNPN-----------  
TPHA0N01420    NRII-----ETEDDINERALSLLQRNRERRRLLQRQ-T---DR-QQFEKILSRKE-----------  
Kpol\_181.3     NRLT-----IEQDSINERALSLLQRNRERRRLLQRR-E---DR-NRYYRADSPDQVTR--------  
ZYRO0F01144g   SV--------EQERINERASQLLQRNRERRRLLQRQQ----ER-RRYEGPPRSLI-----------  
TDEL0B06850    NRLS-----VEQQRINERALSLLQRNRERRELLRRQ-Q---DR-RRFERIPERQ------------  
KLLA0C12529g   GASLI-----DRESINQRALQLLQRNRRRRLLLNRS-E---DK-ARYIQPER--------------  
ABR083C        EQLG-G-F-QNNESINQRALLLLQRNRQRRQLLQRQ-E---DR-TRYIPDDAKRR-----------  
Ecym\_2307      RLSP-----ADNDSINQRALQLLQRNRQRRQLLQRQ-E---DR-LRYVSTKSEHK-----------  
SAKL0B11198g   NRLG-A-QEPSQQSINERAISLLQRNRARRKMLQRQ-E---DR-ERYVRRNELPE-----------  
KLTH0D08602g   NRL--------SRDINERAISLLQKNRQHRELLQRRGE---DR-RRYVPSEEPS------------  
Kwal\_26.8328   NRL--------AQDINEKALSLLQKNRHRRELLQRRGE---DR-RRYVPSEEPS------------  
  
cons                         ::.:\*  :\*::.     ::                                   
  
  
YKL049C        DYEDQAG---------N----LEIETENEEEA--------------------------------EM  
Smik\_11.199    DYDGQAD---------D----LEIETENEEET--------------------------------EV  
Skud\_11.180    NHEGQAE---------N----LETETENEDER--------------------------------EM  
Suva\_11.177    DYEDHAG---------N----LETEAENENET--------------------------------EV  
CAGL0M13145g   DYYHRNQLP------SSYDAGNDDF-EPINNS--------------------------------HV  
KAFR0I01590    RSEDRRRY-------EH----VDMDIIGDEKG---------------------------------M  
KNAG0H00580    PYENELDI---SHVPDD----FDEEFEDRSESEVLSTVNGT--------------------NSIIA  
NCAS0H03050    VASSHYRSNDVGGNFQF----FDQEEDEDEEGNAIDDDYGTLD--------------QSNIIDRHQ  
NDAI0C00590    VISGISEF-------NN----AEEEYFGDQAGNIDDYNFQHYDGEEDISSLEHPEPEHKQIRSRKL  
TBLA0G01890    ----SNPNSNSNPYPYP----NPNTHRGDDSN-D---NGDS--N-------------DDSDQEYIT  
TPHA0N01420    -----IPVAGDVGDDLD----SDREYAGDDGSASDSEVERGLI-DDSASD-------SDSDSDYDT  
Kpol\_181.3     ESIGNEGYNDVEEGEVN----EEIPHVLPSRM------------------------------RYGY  
ZYRO0F01144g   ----------------------NHDYGAPSKN----------------------------------  
TDEL0B06850    ----YIQPA-------------EHRYALDE------------------------------------  
KLLA0C12529g   ------------------------SASSQQIHPP--------------------------------  
ABR083C        ----------------V----VERVAEPAAAESR--------------------------------  
Ecym\_2307      ---------------EN----YERHKANDDARAS--------------------------------  
SAKL0B11198g   --------------------------EQPP------------------------------------  
KLTH0D08602g   ----------------------QIDISAP-------------------------------------  
Kwal\_26.8328   ----------------------HIEQVEPE------------------------------------  
  
cons                                                                               
  
  
YKL049C        ETEVPAPVRTHSYALDRYVRQKRREKQRKQSLKR--VEKKYTPSELALYEIRKYQRSTDLLISKIP  
Smik\_11.199    ATEVSTSARTHSYALDRYVRQKRREKQRKQGLKR--IEKKYSPSELALYEIRKYQRSTDLLISKIP  
Skud\_11.180    ETEVPDATRTHSYALDRYVRQKRRQKQMKQGLKR--VEKKYSPSELALYEIRKYQRSTDLLISKIP  
Suva\_11.177    EEELSTAKQTHSYALDRYVRQKRRQKQRKQGLKR--VEKKYSPSELALYEIRKYQRSTDLLISKIP  
CAGL0M13145g   ESEEENKRLPEKYSLDKYVKRSRKQRDHRHIVAKPKEKRNFAPSKLAMYEIEKYQRSTALLIQKIP  
KAFR0I01590    NNKEKKKKKKKKKRHEKKIESK-IRKIQKERSK----EKKYTPSSLALYEIRKYQGSTDLLISKIP  
KNAG0H00580    PRSVETRRYQRPNKFGQLEKKIKDIQRKRIDGTAAQQGKKFRPSNLALYEIRKYQQSTDLLISKIP  
NCAS0H03050    ERRKGNKHSRQERHHQRELKQR-VEKIRTQRQVG--NTKKFTPSSLALYEIRKYQRSSELLISKIP  
NDAI0C00590    NSRVSKPLRSHDRQHQRRLEEK-AEQIREDKER----RAKYTPSTLALYEIRKYQRSTELLISKIP  
TBLA0G01890    PATTKTKLKPIPNATYSPPHRSRLQELRAIAKTRKNVRKIFTPSQLAEYEIKKYQRSTELLMSKIP  
TPHA0N01420    HMRRNETVERVKQEMSSKKKSKKLR-NKNANP----KKK-FTPSELAMYEIRKYQRSTDLLISKIP  
Kpol\_181.3     DLEEKLRHSRAGNSQGASVTRSRNRVKSKNVMRNPKTGKAFNASELAMYEIRKYQRSTELLISKIP  
ZYRO0F01144g   ------V-EENREQERTQIRQRRQRIRSSHKLS----QKRRTPSDQALYEIRKYQRSTELLISKIP  
TDEL0B06850    -----V--TRKRDELGRQQRKRKVRVNKKVSSHS-----KHTPSELALYEIRKYQRSTELLISKIP  
KLLA0C12529g   ------EHHISAHER--ITKARGT---------------RYKPTDLALAEIRKYQRSTDLLISRMP  
ABR083C        ------ADGAREEER--PARPARPRETRRIAK----KPQRYRPSDVALQEIRRYQRSTELLISRMP  
Ecym\_2307      ------EQRHSEVDGKGILKAKAKRHKSAMTV----KRMRYRPSDVALQEIRKYQRSTELLISRMP  
SAKL0B11198g   ----------------TAIGTIRSNIKKSKSQ----R-KRYKPSDVALQEIRKYQRSTELLISKMP  
KLTH0D08602g   -----------------AVTQIRRRKAST---------KRYRPSDTALQEIRKYQRSTDLLISKMP  
Kwal\_26.8328   -------------TSIVAVPHLRQRKRNT---------KRYRPSDTALQEIRKYQRSTDLLISKMP  
  
cons                                                     .:  \*  \*\*.:\*\* \*: \*\*:.::\*  
  
  
YKL049C        FARLVKEVTDEFTTKDQDLRWQSMAIMALQEASEAYLVGLLEHTNLLALHAKRITIMKKDMQLARR  
Smik\_11.199    FARLVKEVTDEFTTKDQDLRWQSMAIMALQEASEAYLVGLLEHTNLLALHAKRITIMKKDMQLARR  
Skud\_11.180    FARLVKEVTDEFTTKDQDLRWQSMAIMALQEASEAYLVGLLEHTNLLALHAKRITIMKKDMQLARR  
Suva\_11.177    FARLVKEVTDEFTTKDQDLRWQSMAIMALQEASEAYLVGLLEHTNLLALHAKRITIMKKDMQLARR  
CAGL0M13145g   FAKLVKEVTEEFAGESQDLRWQSMAILALQEASEAYLVGLLEHTNLLALHAKRITIMKKDMQLARR  
KAFR0I01590    FARLVKEVSDEFTYRDENLHWQSMAIVALQEASEAYLVGLLEHANLLALHAKRVTLMKKDVQLARR  
KNAG0H00580    FARLVKEVASDFVWESEPLTWQSMAILALQEASEAYLVGLLEHANLLALHAKRVTVTKKDIQLARR  
NCAS0H03050    FTKLVKEVTDEFTVEDQQLHWQSMAIVALQEASEAYLVGLLEHANLLAIHAKRITLMKKDIQLARR  
NDAI0C00590    FTKLVKEVTDQFTVEEQQLHWQSMAIVALQEASEAYLVGLLEHANLLALHAKRITLMRKDIQLARR  
TBLA0G01890    FARLVKEVTDEFTLDEQQFRWQSMAILALQEASEAYLVGLLDHTNLLALHARRITIMKKDMQLARR  
TPHA0N01420    FARLVKEVTEQFTTEEQNFRWQSMAILALQEASEAYLVGLLEHTNLLALHARRITVMRKDMQLARR  
Kpol\_181.3     FARLVKEVTEQFTTEEQQLRWQSIAILALQEASEAYLVGLLEHTNLLALHAKRITVMRKDMQLARR  
ZYRO0F01144g   FARLVREVTEQFTTEEQQPRWQSMAVLALQEASEAYLVGLLEHTNLLALHAKRITIMRKDMQLARR  
TDEL0B06850    FARLVKEVTDQFTTEEQQLRWQSMAILALQEASEAYLVGLLEHTNLLALHAKRITIMRKDMQLARR  
KLLA0C12529g   FARLVKEVTDQFTTESEPLRWQSMAIMALQEASEAYLVGLLEHTNLLALHAKRITIMRKDMQLARR  
ABR083C        FARLVKEVTDQFTTVDQQMRWQSMAILALQEASEAYIVGLLEHTNLLALHAKRVTVMRKDMQLARR  
Ecym\_2307      FARLVKEVTDQFTTEEQQLRWQSMAILALQEASEAYLVGLLEHTNLLALHAKRITIMRKDMQLARR  
SAKL0B11198g   FARLVKEVTEQFSTDEQQLRWQSMAILALQEASEAYLVGLLEHTNLLALHAKRITIMKKDMQLARR  
KLTH0D08602g   FARLVKEVTDQFTTEEQQLRWQSMAIMALQEASEAYLVGLLEHTNLLALHAKRITIMRKDMQLARR  
Kwal\_26.8328   FARLVKEVTDQYTTEEQQLRWQSMAIMALQEASEAYLVGLLEHTNLLALHAKRVTIMRKDMQLARR  
  
cons           \*::\*\*:\*\*:.::   .:   \*\*\*:\*::\*\*\*\*\*\*\*\*\*:\*\*\*\*:\*:\*\*\*\*:\*\*:\*:\*: :\*\*:\*\*\*\*\*  
  
  
YKL049C        IRGQFI  
Smik\_11.199    IRGQFI  
Skud\_11.180    IRGQFI  
Suva\_11.177    IRGQFI  
CAGL0M13145g   IRGQFI  
KAFR0I01590    IRGQFI  
KNAG0H00580    IRGQFI  
NCAS0H03050    IRGQFI  
NDAI0C00590    IRGQFI  
TBLA0G01890    IRGQFI  
TPHA0N01420    IRGQFI  
Kpol\_181.3     IRGQFI  
ZYRO0F01144g   IRGQFL  
TDEL0B06850    IRGQFL  
KLLA0C12529g   IRGQFI  
ABR083C        IRGQFI  
Ecym\_2307      IRGQFI  
SAKL0B11198g   IRGQFI  
KLTH0D08602g   IRGQFI  
Kwal\_26.8328   IRGQFI  
  
cons           \*\*\*\*\*:  
  
  
  
  
  
