# Supplementary material for: Loss of inner kinetochore genes is associated with the transition to an unconventional point centromere in budding yeast
Source: PeerJ. 2020 Sep 29;8:e10085. doi: 10.7717/peerj.10085 (PMC7531349; doi:10.7717/peerj.10085)
Supplement: Supplemental Information 3 — The multiple sequence alignment of open reading frame nucleotide sequences and amino acid sequences generated using MUSCLE, M-coffee, Clustal Omega and Guidance with PRANK are provided along with the commands used for the command line tools. Results of the selection tests from BUSTED, FEL, MEME and RELAX from the HYPHY package are provided as json files that can be visualized using the online hyphy vision website. [file peerj-08-10085-s003.zip › Supplementary_Material_S3/ORFs/M-Coffee_result_CSE4_ORFs.score_html]

T-COFFEE, Version\_11.00.d625267 (2016-01-11 15:25:41 - Revision d625267 - Build 507)  
Cedric Notredame   
CPU TIME:0 sec.  
SCORE=594  
\*  
 BAD AVG GOOD  
\*  
YKL049C        :  42  
Smik\_11.199    :  41  
Skud\_11.180    :  41  
Suva\_11.177    :  42  
CAGL0M13145g   :  31  
KAFR0I01590    :  36  
KNAG0H00580    :  46  
NCAS0H03050    :  33  
NDAI0C00590    :  31  
TBLA0G01890    :  36  
TPHA0N01420    :  36  
Kpol\_181.3     :  45  
ZYRO0F01144g   :  43  
TDEL0B06850    :  42  
KLLA0C12529g   :  45  
ABR083C        :  40  
Ecym\_2307      :  47  
SAKL0B11198g   :  43  
KLTH0D08602g   :  44  
Kwal\_26.8328   :  43  
cons           :  59  
  
YKL049C        ATGTCAAGTAAACAACAATGGGTTAG-----TTC----------T--------------G-CTATT  
Smik\_11.199    ATGTCAAGCAAACAGCAATGGGCTAA-----TTC----------T--------------G-CTATT  
Skud\_11.180    ATGTCAAGCAAACAGCAATGGGCTAA-----TTC----------G--------------G-CCATT  
Suva\_11.177    ATGTCTAGCAAACAAGAATGGGCTAA-----CTC----------G--------------G-CCATT  
CAGL0M13145g   ATGTCTACTAGGCAAGCGGTG---------------------------------------------  
KAFR0I01590    ATGCAGAGTCAGCAATG-------------------------------------------------  
KNAG0H00580    ATGCAGAAACAACAAT------------------------------------------------TT  
NCAS0H03050    ATGTCAAGCAGAAAATTTGTAGAACAAGGCCATGCTCAGAATTCTAGTAGTCATCTGTTCTC----  
NDAI0C00590    ATGCAGAGTAGGAAATGGATCCCATCAGATCGTGGCAACGAATCAACTAGCCATCTTTTCTCTAGC  
TBLA0G01890    ATGTCGC-----------------------------------------------------------  
TPHA0N01420    ATGGAGACAAATGGATC-------------------------------------------------  
Kpol\_181.3     ATGTTGAG-----------------T------AG----------C---------------AGGATT  
ZYRO0F01144g   ATGGAGACTCGACAACT-----------------GGA----TG-----------------------  
TDEL0B06850    ATGGAGCTCAAACAGTTGGACCACAA--C---GC----------C--------------A-GCATC  
KLLA0C12529g   ATG------GAACAGT--CTA-------------------------------------------TC  
ABR083C        ATGGAAC-AGAGC--------------------------------------------------ATG  
Ecym\_2307      ---------------ATGGAACAAA------------------------------------GTATA  
SAKL0B11198g   ATGGATAGACAAGAATGGA------A-----TGC----------T---------------AGTATA  
KLTH0D08602g   ATGAACGCC-A--------------------------------------------------GCATA  
Kwal\_26.8328   ATGAACGCA-AGTA--------------------------------------------------TA  
  
cons                                                                               
  
  
YKL049C        C--AAA-------------------GTG-----------ATTCGAGT--GGA-AGATCA-------  
Smik\_11.199    C--AAA-------------------GTG-----------ATTCAAGT--GGA-AGATCA-------  
Skud\_11.180    C--AAA-------------------GTG-----------ATTCGAGT--GGA-AGGTCA-------  
Suva\_11.177    C--AAA-------------------GTG-----------AATCGAGT--GGA-AGATCA-------  
CAGL0M13145g   TTTGAA-------------------AGAGATGTGGATGAAGATAGATGGCCC-AGATCA-GCA-CG  
KAFR0I01590    ---GGA---------------------------------------GC----------AG-------  
KNAG0H00580    G--ATA-------------------GTA-----------GCGATTGGG--AT-CT-GTT-------  
NCAS0H03050    ------------------------------------------------------------------  
NDAI0C00590    G--TAA-ACAACAGCGCTAATAATAATT-----------ATGAAAGA--GCA-AGATCGATACATA  
TBLA0G01890    ------------------------------------------------------------------  
TPHA0N01420    ----------------------------------------------A--AAT-AGGACA-------  
Kpol\_181.3     C--CAA-------------------ACA-----------ATTCTGGGA--AT-CGAGCA-------  
ZYRO0F01144g   ------------------------------------------------------------------  
TDEL0B06850    C--ACA-------------------GCG-----------ACTCTGGT--GCT-AGGACATTGAGCA  
KLLA0C12529g   A--GGA-------------------GCA-----------TAGACGGT--TCT-AGATCT-------  
ABR083C        T--CAA-------------------GCG-----------AGCAGGCTG--AGCGGGTGG-------  
Ecym\_2307      C--GTA-------------------ACG-----------AGCTGCCT--AGC-AGGAAT-------  
SAKL0B11198g   T--CCA-------------------GCC-----------AGACAGG-C-----AGATCA-------  
KLTH0D08602g   G--CAAGC-------------------C-----------AAACAGGT--GGC-AGATCG-------  
Kwal\_26.8328   G--CGA-------------------GTC-----------AGGTTGGGG--GG-AGATCA-------  
  
cons                                                                               
  
  
YKL049C        -----------------------------------------------CTCAG---T-AACGTCAAC  
Smik\_11.199    -----------------------------------------------CTCAG---C-AATGTCAAC  
Skud\_11.180    -----------------------------------------------CTCAG---C-AATGTGAAC  
Suva\_11.177    -----------------------------------------------CTCAG---T-AATGTCAAT  
CAGL0M13145g   --------------------AGGG-----------------------TTGGC---T-GGTGTTAAC  
KAFR0I01590    -----------------------------------------------TC------G-AATGTGAAT  
KNAG0H00580    -----------------------------------------------GCCCACCGC-GCCGCTGAC  
NCAS0H03050    -------------------GAATATCCTCGATAATGATAGTGGATCATTGAG---C-AATATAAAT  
NDAI0C00590    ATGTGAATGGATTACTCCCGAGTATAATAGCACATAGGATTAACGAAGAGGA---TGAAGATGAAG  
TBLA0G01890    ---------------------------------------AACGCACCATCAG---C-AATATAAAC  
TPHA0N01420    -----------------------------------------------TTGAC---T-AATGTCAAT  
Kpol\_181.3     -----------------------------------------------TTGAG---T-AATATAAAT  
ZYRO0F01144g   ------------------------------GTGCAGTTCACAATGGCTCAAG---A-CTCTTAAGT  
TDEL0B06850    ACGTGAACA---------------------------------------------------------  
KLLA0C12529g   -----------------------------------------------TTGAG---C-AATGTTGGC  
ABR083C        -----------------------------------------------CCGGG---C-GGGTGGAGC  
Ecym\_2307      -----------------------------------------------TT--------AG-TTAGGC  
SAKL0B11198g   -----------------------------------------------CTCAG---T-AACACCAAT  
KLTH0D08602g   -----------------------------------------------TTGAGC----AATACGAAT  
Kwal\_26.8328   -----------------------------------------------CTCAG---T-AATACCAAC  
  
cons                                                                               
  
  
YKL049C        AG---------------------------------GCTTG----C-A-GG---------AGACCAA  
Smik\_11.199    AG---------------------------------ACTTG----T-T-GG---------AGAGCAA  
Skud\_11.180    AG---------------------------------GCTAG----T-A-GA---------AGATCAA  
Suva\_11.177    AG---------------------------------GCTTG----C-A-GG---------AGATCAA  
CAGL0M13145g   A---------------------------------CTGTAT----T-T-GA---------AGGTGAT  
KAFR0I01590    A------------------------------GAGGTTTGC----T-C-GA---------GGAGCAA  
KNAG0H00580    TA---------------------------------C-----------------------TGACCAG  
NCAS0H03050    AGATTAACACTGGACCCGGATAATACAGA---AG-ATTTA----T-T------------ACAGCAA  
NDAI0C00590    AAGAAGATGC-AGATTTTGATGACCTTGATGCAG-ATTTA----C-GTCT---------TGAACAA  
TBLA0G01890    AGAC---------------------------------TTA----A-C-GA---------TGAACAG  
TPHA0N01420    A---------------------------------GGATCA----T-A-GA---------GACTGAA  
Kpol\_181.3     AG---------------------------------GCTTA----C-T-AT---------TGAACAG  
ZYRO0F01144g   CAGT---------------------------------TAT----C-T-GT---------AGAGCAA  
TDEL0B06850    ---------------------------------G-GTTAT----C-T-GT---------AGAACAA  
KLLA0C12529g   G---------------------------------CTTCAC----T-T-AT---------AGATCGT  
ABR083C        AG---------------------------------CTTGGAGGGTTC-CA---------GAACAAC  
Ecym\_2307      TT---------------------------------AGT-C----C-T-GC---------CGATAAT  
SAKL0B11198g   CG---------------------------------TCTGG----G-C-GCACAAGAGCCCTCTCAA  
KLTH0D08602g   CG---------------------------------ACTCT----C-T-A-----------------  
Kwal\_26.8328   AG---------------------------------A-----------------------CTGGCGC  
  
cons                                                                               
  
  
YKL049C        CAA---TCTATTAACGATCGTGCGTTATCGTTATTGCAGAGAACAAGAGCGACAAAGAACCTGTTT  
Smik\_11.199    CAA---TCCATTAACGATCGTGCATTATCATTATTGCAAAGGACAAGAGCAAGAAAGAATCTATTT  
Skud\_11.180    CAG---TCTATTAATGACCGTGCACTATCTTTGTTACAAAGAACAAGAGCAAGAAAAAATCTTTTT  
Suva\_11.177    CAG---TCGATTAATGATCGTGCGTTATCCTTGTTACAAAGAACAAGAGCAAGGAAGAACCTGTTC  
CAGL0M13145g   ---TCAGAGATTAACTCTAAAGCAATGCGATTATTAGAGAAGACTAGGCATCGCAGGAATTTACTA  
KAFR0I01590    GAGTT---GATCAATGAGAGGGCGAATTTGTTATTACAGAGGACGAGAGAACGGAGGAATCTGTTG  
KNAG0H00580    GAG---AAAATCAATGAGAGAGCGCGGGAACTTCTGGAGAGAAACCGGAGGTACGGGAGACTGCTG  
NCAS0H03050    GAA---GTCATCAACGAGCGGGCACTTTCTCTTTTACAAAGGACTAGAGAACGTAGAAACTTATTA  
NDAI0C00590    GAT---GACATTGATCAAAAGGCACTTTTATTGTTACAAAGGACAAGAGAACGTAAAAATTTATTG  
TBLA0G01890    ACT---CTTTTGAACCAAAGAGCTGCCCAGTTCTTACAACGTAACATCCAAGGGAGAAGACTGTTC  
TPHA0N01420    GATGA---TATAAATGAACGAGCTCTATCGTTGTTGCAACGTAATAGAGAGAGGAGGAGGTTGCTA  
Kpol\_181.3     GAC---TCTATTAATGAGAGAGCTTTGTCTCTGTTGCAGCGTAATAGAGAGAGGAGAAGACTTTTG  
ZYRO0F01144g   GAG---CGAATTAACGAAAGGGCATCTCAGCTGCTGCAGCGTAATAGAGAGCGTAGGAGGTTATTA  
TDEL0B06850    CAG---CGGATTAATGAAAGAGCCCTATCACTCCTTCAACGTAATAGAGAACGTAGAGAGTTGCTA  
KLLA0C12529g   GAATC---GATCAATCAGAGAGCACTACAATTATTACAGAGAAACAGAAGAAGACGTTTGTTGTTA  
ABR083C        GAG---TCAATCAACCAGCGGGCGCTGCTGCTGCTGCAGCGCAACCGGCAGCGGCGACAGCTGCTA  
Ecym\_2307      GAT---TCGATAAACCAAAGGGCACTACAACTATTACAGAGAAATCGACAGAGAAGACAGTTACTT  
SAKL0B11198g   CAA---TCTATCAACGAGAGGGCAATATCGCTTCTACAGCGTAATAGGGCCAGGAGGAAGATGTTA  
KLTH0D08602g   GAG---A-CATCAACGAAAGGGCAATATCGCTTTTACAGAAAAACCGGCAGCACAGGGAGCTGTTG  
Kwal\_26.8328   AAG---A-TATCAATGAGAAAGCATTGTCACTACTTCAGAAGAATAGGCATCGTAGGGAGCTGCTT  
  
cons                     \*  \*       \*\*        \*  \*  \*    \*                  \*  \*   
  
  
YKL049C        CCAAGAA---GA------------GAGGAAAGAAGACGTTATGAAA---GCTCA---AAA------  
Smik\_11.199    CCAAGAA---GA------------GAAGAAAGGAGACGATATGAGA---ACCCA---AAG------  
Skud\_11.180    CCAAAAA---GA------------GAAGAAAGAAGACGATATGAAG---GTCCA---CAA------  
Suva\_11.177    CCAAGAA---GA------------GAAGAAAGAAGACGGTATGAAG---GTTCA---CAG------  
CAGL0M13145g   AATCGTC---GA------------GAGGATCGTCGCCGTTATTTGG---GAGGG---GTT------  
KAFR0I01590    TTGCAAG---GT------------ACGGGAGAGCATGGGAATTTAC---TGTTA---CAA------  
KNAG0H00580    AAAACGG---GCGGCGATCCGCTCCCAGAGCGAAGGCGGTACGAAAGAGACCTG---GA-------  
NCAS0H03050    CACCGAT---TT------------GAAGATAAAAGAAGGTATTATA---ACCAAGGGCAA------  
NDAI0C00590    AATGGAC---AATTGCCACCGGAAGAAGATAGAGGAAGATATTATC---AAAAT---ACT------  
TBLA0G01890    CAACGAT---AC------------AAGGAAACCAAACGGTTCGAGA---ACTCA---AAC------  
TPHA0N01420    CAGAGAC---AG------------ACTGATCGGCAACAGTTTGAGA---AAATA---CTG------  
Kpol\_181.3     CAAAGAA---GG------------GAAGACAGGAATAGGTACTACC---GTGCT---GATTCTCCA  
ZYRO0F01144g   CAACGTC---AA------------CAAGAAAGAAGGCGATACGAGG---GCCCA---CCA------  
TDEL0B06850    AGGAGGC---AA------------CAAGATAGACGAAGGTTTGAAC---GAATA---CC-------  
KLLA0C12529g   AATAGAA---GT------------GAAGACAAAGCAAGATACATCC---AGCCC---G--------  
ABR083C        CAGAGACA---G------------GAAGACCGCACACGGTACATAC---CGGAC---GAC------  
Ecym\_2307      CAAAGGC---AG------------GAGGACAGGCTACGTTATGTTT---CCACC---AAG------  
SAKL0B11198g   CAAAGGC---AA------------GAGGATAGAGAGCGATACGTAA---GGC--------------  
KLTH0D08602g   CAGCGGCGAGGA------------GAAGACCGCAGAAGATATGTTC---CAT-C-----A------  
Kwal\_26.8328   CAGAGGA---GG---------GGAGAAGATCGCAGGCGGTACGTTC---CC---------------  
  
cons                                      \*                                        
  
  
YKL049C        -----AGTGACCTAG--ATATCGAAACAGAC---TA---------------CGAAGACCAAG----  
Smik\_11.199    -----GATGACGTAG--ATTTTGAAACTGAC---TA---------------TGATGGCCAAG----  
Skud\_11.180    -----GATGACATGA--TTTTTGAAGAAAAC---CA---------------TGAAGGCCAAG----  
Suva\_11.177    -----GATGACGTAG--AATTTGAGGCAGAT---TA---------------CGAAGACCATG----  
CAGL0M13145g   -----AAGGCAAAA--GCAAT--T---------------------------G------AAT-CAG-  
KAFR0I01590    -----GGTACAACA--GATTTGTTGCCCCGTTCAGAGGATA----------G------AAG-GCG-  
KNAG0H00580    -------CTCAACA-----GATATAC------AGGATGGGGACGTTGTCTCCAATAGTGGTCCGTA  
NCAS0H03050    -----GACGACGGAG--ATTTAGAGTCTGTGGCCAGTAGTCATTATCGTTCTAATGATGTGGGTGG  
NDAI0C00590    -----ATGAATGATG--ATATAGGATCTGTCATAAGTGGGATCAGTGAATTCAATAATGCGGAGGA  
TBLA0G01890    -----TCAAACCCAA--ACTCAAACCCAAACTCAAACTCAAACCCATACCCATACCCAAACCCAAA  
TPHA0N01420    -----TCGAGAAAAGAGA--TACCCGTTGCCGGGGATGTGGGTGATGACTTGGATTCAGATAGAG-  
Kpol\_181.3     GATCAGGTGACGAGAG---AATCCATTGGAA-----A--------------TGAAGGGTATA----  
ZYRO0F01144g   -----C-----GGTC--ATTGATCAATCATGACT---------A--------TG--GT--------  
TDEL0B06850    ------TGAACGG-----------------------------CAATATATTCAAC-CTGCGGAG--  
KLLA0C12529g   -----------A---------------------------------------G--------------  
ABR083C        -----GCGAAAC----------GA--CGGGT---------------------GG---TGGAG----  
Ecym\_2307      -----AGTGAGC--------ATAAAGAGAAT---TA---------------TGAAAGGCATA----  
SAKL0B11198g   ------------------------------------------------------------------  
KLTH0D08602g   -----G------------------------------------------------------------  
Kwal\_26.8328   ------------------------------------------------------------------  
  
cons                                                                               
  
  
YKL049C        --------------CAGGTAA--TC---TAGA---AA-T---CGAGA---C--AGA-AAA------  
Smik\_11.199    --------------CAGATGA--TT---TGGA---GA-T---AGAAA---C--TGA-GAA------  
Skud\_11.180    --------------CGGAAAA--CT---TGGA---AA-C---AGAGA---C--AGA-AAA------  
Suva\_11.177    --------------CAGGAAA--TC---TCGA---AA-C---TGAAG---C--AGA-AAA------  
CAGL0M13145g   --ATT---ATTACCAT--CGC--AATCAACTA-CCAA-GCTCAT------A--TGA----------  
KAFR0I01590    --AT-------AT-----GAG--CA-TGTGGA-TATG-GA---T------A--TAA---TT-----  
KNAG0H00580    CGAGA---ACGAG-CTCGAC---------A-TAT-CGCACGTTCCGGACGA--TTT-CGAT-----  
NCAS0H03050    GAACTTTCAATTTTTTGATCAAGAAGAAG------AT-GA---GGAT---GAGGAAGGT-A-----  
NDAI0C00590    GGAAT---ATTTTGGTGAC-----CAGGC------CG-GC---AATA---T--CGA-TGATTATAA  
TBLA0G01890    CACA---CATA-GAG--GCGA--CGATTC------CA-AC---GACA---A--CGG-TGAT-----  
TPHA0N01420    --AGT------ATGCTGGAGA--T---GATGG-C------AGTGCAA---G--TGA-CAGT-----  
Kpol\_181.3     --------------ACGATGT--TG---AAGA---AG-G---AG-AA---G--TAA-ATG------  
ZYRO0F01144g   -GCTCC-TA--GCA----------AGA--------AC-GT---GGAA---GA--------------  
TDEL0B06850    ----C----------------------AC------CG-G----T-----------A-CG-------  
KLLA0C12529g   ---------------------------------------C--------------GT----------  
ABR083C        --------------AGAGTTG--CG---GA------------------------------------  
Ecym\_2307      --------------AGGCGAA--TG---ATG--------------AT---G--CTA-GAG------  
SAKL0B11198g   -----------------------------------------------------------G------  
KLTH0D08602g   -------------------------------------------------------A-AGAA-----  
Kwal\_26.8328   ----------------------------------------------TCC-----GA-AGAG-----  
  
cons                                                                               
  
  
YKL049C        ----------------TGAAGAAGAAGCT-------------------------------------  
Smik\_11.199    ----------------CGAAGAAGAAACT-------------------------------------  
Skud\_11.180    ----------------CGAGGATGAACGT-------------------------------------  
Suva\_11.177    ----------------CGAAAATGAAACA-------------------------------------  
CAGL0M13145g   ------------TGCTGGTAACGA--CGATT-TTGAGC--------CGATCAAT---AA--C-TC-  
KAFR0I01590    -----G------GTGACGAGA--------AG-GG--GA--------TGAACAAC------------  
KNAG0H00580    -----------------GAAGAATTTGAA-------------------------------------  
NCAS0H03050    ---ACGCCATTGATGACGATTATGGAACCCTAGA-T--CA----ATCCAA---CA-T--TATC---  
NDAI0C00590    TTTCCAAC-ATTATGACGGAGAAGAAGATATATC-T-TCT----CTCGAA---CA-T--CCTG---  
TBLA0G01890    -----TCT---AATGATGATTCAGAC--CAAGAA-T-ATATTACTCCAGC---CA-----------  
TPHA0N01420    -----G------AGGTGGAGAG----AG-GT------C--------TAATTGATGACAGTGC-AAG  
Kpol\_181.3     ----------------AGGAGATCCCACA-------------------------------------  
ZYRO0F01144g   ----------------AAATAGGGAA--CAGGAA-A-----------GA-----------------  
TDEL0B06850    --C-------------C-----T------------------------------TA-G--ATGAAG-  
KLLA0C12529g   -------------T------------------------------------CTGC------------  
ABR083C        ------------------------------------------------------------------  
Ecym\_2307      ----------------CTA-----------------------------------------------  
SAKL0B11198g   ----------------T-------------------------------------------------  
KLTH0D08602g   -----------------CCTTCTCAAATT-------------------------------------  
Kwal\_26.8328   -----------------CCTTCACACATT-------------------------------------  
  
cons                                                                               
  
  
YKL049C        ---------------------GAAA--TG---GAAACTG-AAG--------TAC-------C---T  
Smik\_11.199    ---------------------GAAG--TG---GCTACTG-AAG--------TAT-------C---A  
Skud\_11.180    ---------------------GAAA--TG---GAAACAG-AGG--------TTC-------C---A  
Suva\_11.177    ---------------------GAAG--TA---GAAGAAG-AAT--------TAT-------C---G  
CAGL0M13145g   --------------------ACATG--TT---GAATCAG-AAG--------AAG-------A---A  
KAFR0I01590    ----------------------AAGG--A---GAAGAAG-AAG--------AAG-------A----  
KNAG0H00580    ---------------------GACA--GATCTGAATCAG-AGG--------TCTTAAGTACC---G  
NCAS0H03050    GA-TCG--TC---ACCAAGA----------------AAG-AAG--------AAA-------AGGAA  
NDAI0C00590    AA-CCA-GAA--CATAAACA----------------GAT-AAG--------AAG-------TAGAA  
TBLA0G01890    CCACCA-AGA--CTAAACTG-----------------AAACCA--------ATA-------CCCAA  
TPHA0N01420    TG-ACA-G-T--GACA-GCGACAG----T---GATTACG-A-T--------ACG-------C----  
Kpol\_181.3     ---------------------TGTA--TT---GCCATCA-CGGATGAGGTATGG-------T---T  
ZYRO0F01144g   -------------------------------------A--CTC--------AAA-------TT--A  
TDEL0B06850    TA-ACAAGAAAGCG-CGATG----------------A-G-CTG--------GGG-------AG---  
KLLA0C12529g   ----------------------TAGCAGC---CA-G-CA-GA----------TT-------C---A  
ABR083C        ----------------------------------------------------GCC----------G  
Ecym\_2307      ------------------------------------------------------------------  
SAKL0B11198g   ---------------------------------------------------AAT-------G---A  
KLTH0D08602g   ---------------------GATA--TATCTGCACCT----------------------------  
Kwal\_26.8328   ---------------------GAAC--AGGTTGAGCCAG-AAA--------CTTCAA---------  
  
cons                                                                               
  
  
YKL049C        G---------CACCA--GTG-CGAA-----------CTC-ATTCATATG---CC------------  
Smik\_11.199    A---------CGTCC--GCG-CGAA-----------CAC-ATTCGTATG---CC------------  
Skud\_11.180    G---------ACGCT--ACC-CGAA-----------CAC-ATTCATACG---CT------------  
Suva\_11.177    A---------CGGCT--AAG-CAAA-----------CTC-ACTCGTACG---CC------------  
CAGL0M13145g   A---------ATAAG--AGA-CTT-----------CCTG-AGAAATACT---CT------------  
KAFR0I01590    ----------AGAAG--AAG-AAG------------AAG-AG------A---CA------------  
KNAG0H00580    T---------TAACG--GTA-CAAA-----------TTC-CATAATAGC---GCCGCGGTCCGTCG  
NCAS0H03050    A--C-----AAACAT---TCTCGTCAAGAAA-GACACCAT--------------------------  
NDAI0C00590    AACTGAATAGTAGAGTCAGT-AAACCCTTGA-GGAGCCATG-AT---CGACAAC------------  
TBLA0G01890    T--G-----CCACATACAGTCC-TCCTCA-------TCGTT-C-----A-----------------  
TPHA0N01420    ----------ACATG--AGA-AGAAAT-GAGACG-GTAG-AGC-----G---AG------------  
Kpol\_181.3     A---------TGATT--TGG-AAGA-----------GAA-ACTTCGACA---CT------------  
ZYRO0F01144g   ---G-----ACAGAG---------------------------------------------------  
TDEL0B06850    -----------------------G-------------CAAC-AG---AGG-AAG------------  
KLLA0C12529g   C---------CCTC----------------------CAG-AG------------------------  
ABR083C        G---------CGGCT--GCG-GAAT-----------CGC-GTGCGGACG---GC------------  
Ecym\_2307      G---------TGAGC-------AAA-----------GAC-ATTC----------------------  
SAKL0B11198g   A---------C--------------------------TA-C-------------------------  
KLTH0D08602g   -----------------G------CT-----------GT--------------A------------  
Kwal\_26.8328   ------------------------------------------------------------------  
  
cons                                                                               
  
  
YKL049C        ---------------T--TAGA--CA-GATA-----------T---G------TT-------AG--  
Smik\_11.199    ---------------T--TGGA--CA-GATA-----------C---G------TT-------AG--  
Skud\_11.180    ---------------C--TAGA--TA-GATA-----------T---G------TT-------AG--  
Suva\_11.177    ---------------T--TAGA--TA-GATA-----------T---G------TT-------AG--  
CAGL0M13145g   ---------------T--TGGA--CA-AATA-----------C---G------TT-------AAAC  
KAFR0I01590    ---------------T--GAGA--AA-A-------------------------AG-------ATC-  
KNAG0H00580    AGACTCGTAGATACCAA-AGGC--CA-AATAAGT---------T------TGGCC-------AAC-  
NCAS0H03050    -------------------CAA--A-GA-------------------------GA-------A---  
NDAI0C00590    ---------------A--TCAA--A-GA-------------------------AG-------G---  
TBLA0G01890    -------------------CGATTA-CA-------------------------AG-------A---  
TPHA0N01420    ---------------T--CAAA--CAGGAG-----------------------AT-------GTCA  
Kpol\_181.3     ---------------CTAGGGC--TG-GGAA--TTCCCAA-GGTGCC------TCTGTCACTAGA-  
ZYRO0F01144g   ---------------------------A-------------------------AG-------A---  
TDEL0B06850    ---------------A--GGAA--------------------------------A-------G---  
KLLA0C12529g   ------------------------CA-TC------------------------AT-------ATCA  
ABR083C        -----------------GCGCG--CG-AGGA-----------------------------------  
Ecym\_2307      ---------------------A---G-AAGT-----------TGATGGTAAGGGC-------AT--  
SAKL0B11198g   ----------------------------------CAGAGGAAC----------A-GCCTCCAACT-  
KLTH0D08602g   -------------------ACA--CA-AATT-----------------------C-----------  
Kwal\_26.8328   ------------------------TA-GTGGCAG---------T--------C---------CC--  
  
cons                                                                               
  
  
YKL049C        ACA-GA-AAA------GGAGGG-AAAAA---CAAAG-AA-AGC-AG---AG--------------C  
Smik\_11.199    ACA-GA-AAA------GAAGAG-AAAAG---CAAAG-GA-AAC-AG---GG--------------C  
Skud\_11.180    GCA-GA-AAA------GGAGAC-AAAAA---CAAAT-GA-AGC-AG---GG--------------T  
Suva\_11.177    ACA-AA-AAA------GAAGAC-AAAAA---CAAAG-GA-AGC-AG---GG--------------C  
CAGL0M13145g   --G-TA-GCA------GAAAGCAAAGAG-ATCATAG-GC-ACA-TA---GT---G---G------C  
KAFR0I01590    --G-AATCG--------------AAGA-----TTAG-GA-AAA-TT--CAA---A---A-------  
KNAG0H00580    -TG-GA-GAA------GAAGAT-CAAAG-A---TAT-AC-AGA-GG---AAGAGAATTGACGGAAC  
NCAS0H03050    CTCAAACA------------ACGT---GTTGAAAA-GAT-TAG-AACTCAG---AG--A------C  
NDAI0C00590    TTA-GAA--------------------GA--AAAA-GCC-GAGCAAATCAG---AG--A------A  
TBLA0G01890    GTT-AAGA-------------------GC--CATT-GCC-AAG--ACTC-----------------  
TPHA0N01420    TCA-AA-G--------A----A-GAAAAGCAAG-AAGCT-ACG-AA---AT---A---A------G  
Kpol\_181.3     -TC-GA-GAA------ATAGGG-TAAAATCCA-A---AA-ATG-TA---AT---GA--G------G  
ZYRO0F01144g   CAA-AGGA------------TTA----GA--TCTT-CGC-ATA--AGTT-----------------  
TDEL0B06850    TAC-G----------------------GG--T--C-AAT-AAG-AA---------G--G------T  
KLLA0C12529g   --G-CG-C-----------------AC-----AT---GA-AAG-AA---TA---A---C------A  
ABR083C        --G-GA-GCGGCCCGCGCGTCC-CGCGCGG-CCACG-GGAAAC-GC---GT---CG--G-------  
Ecym\_2307      TTT-AA-AAG------CTAAGG-CAAAA------AG-AC-ATA-AA---TC---T---G------C  
SAKL0B11198g   -GC-GA-TAG------GTACCA-TCAGAAGCAATAT-AA-AAA-AA---TC---C---A------A  
KLTH0D08602g   ------------------------GC---C--GGCG-C--A-A--A--------------------  
Kwal\_26.8328   -TC-AT----------CT-TC---GT---C----------A----A---C---G-GAAA-------  
  
cons                                                                               
  
  
YKL049C        -TTAAAG----CGC-GTCGA-AAAGAAATATACTCCTAGTGAATTAGCTCTGTACGAAATTCGAAA  
Smik\_11.199    -TTAAAG----CGT-ATTGA-AAAGAAGTACAGTCCAAGTGAATTAGCTCTATACGAAATTCGAAA  
Skud\_11.180    -TTGAAA----CGC-GTAGA-AAAGAAATACAGTCCCAGTGAATTAGCTCTGTACGAAATCAGAAA  
Suva\_11.177    -TTGAAG----CGT-GTGGA-AAAGAAATATAGCCCTAGCGAATTAGCTCTTTACGAAATTCGAAA  
CAGL0M13145g   -GA-AGCC---TAA-AGAGAAACGAAACTTTGCTCCTAGTAAGCTTGCCATGTATGAGATTGAAAA  
KAFR0I01590    ----AGAAAGAAGT-AAAGAG-AAGAAGTATACCCCAAGTAGTTTGGCATTGTATGAAATTAGAAA  
KNAG0H00580    TGC-GGCC----CA-ACAGGGGAAGAAATTTAGACCGAGTAATTTGGCTTTGTACGAAATCAGGAA  
NCAS0H03050    --A-GGT----CGGAAAT-ACGAAAAAATTTACTCCAAGTAGTTTAGCCCTATATGAGATAAGGAA  
NDAI0C00590    -GA-TAA----AGA-AAGAAGAGCCAAATATACTCCGAGTACATTAGCATTATATGAAATTAGGAA  
TBLA0G01890    --G-GAA----AAATGTA-AGGAAAATCTTCACCCCGTCACAATTGGCAGAATATGAAATCAAGAA  
TPHA0N01420    -AA-TGC----TAA-TCCTAAGAAGAAATTCACGCCCAGTGAATTAGCAATGTATGAAATCAGGAA  
Kpol\_181.3     -AA-TCC----TAA-AACTGGCAAAGCATTCAACGCTAGTGAATTGGCTATGTATGAAATTAGAAA  
ZYRO0F01144g   --A---T----CA------CAAAAGAGACGTACACCGAGTGATCAAGCTCTTTATGAGATAAGGAA  
TDEL0B06850    -CA-GTT----C------GCATAGTAAGCATACACCGAGTGAATTGGCTCTATATGAAATAAGGAA  
KLLA0C12529g   -AA-GGCT---CGG-GGA----ACTAGATATAAACCTACTGACCTAGCTTTGGCAGAAATAAGGAA  
ABR083C        -AT-AGC----CAA-GAAGCCGCAGCGATACCGGCCCAGCGACGTGGCGCTCCAGGAGATCCGCAG  
Ecym\_2307      AATGACT----GTG--AAGAGGATGAGGTACAGACCTAGTGATGTAGCTTTACAAGAGATCAGGAA  
SAKL0B11198g   -G--AGT----C-----AGCGTAAACGATACAAACCAAGTGATGTGGCTTTGCAAGAGATTAGAAA  
KLTH0D08602g   ----GCG----AGC-ACA----AAAAGGTACCGTCCGAGCGATACAGCGTTACAAGAAATACGCAA  
Kwal\_26.8328   ----CGCA----A--CA---CGAAACGGTATCGCCCCAGTGATACAGCACTTCAGGAAATTCGTAA  
  
cons                                              \*          \*\*       \*\* \*\*    \*   
  
  
YKL049C        ATACCAACGTTCCACGGATTTATTAATCTCCAAAATTCCATTTGCAAGGCTAGTGAAAGAAGTTAC  
Smik\_11.199    GTACCAACGTTCCACGGATTTATTGATTTCCAAAATTCCTTTTGCAAGGCTTGTAAAAGAAGTTAC  
Skud\_11.180    ATATCAACGTTCTACAGATTTGCTTATCTCTAAAATTCCATTTGCAAGACTGGTAAAAGAAGTTAC  
Suva\_11.177    ATACCAGCGTTCCACAGATTTGCTAATCTCCAAAATCCCATTTGCAAGACTAGTCAAGGAAGTCAC  
CAGL0M13145g   ATATCAAAGATCAACAGCATTACTGATACAAAAGATCCCATTTGCTAAACTTGTCAAGGAAGTCAC  
KAFR0I01590    GTATCAGGGATCGACAGACTTACTAATATCAAAGATACCCTTTGCGAGACTTGTGAAGGAAGTCAG  
KNAG0H00580    GTACCAACAATCCACGGACCTTTTAATATCAAAGATACCATTTGCTCGATTGGTCAAAGAGGTTGC  
NCAS0H03050    ATACCAACGATCTTCAGAACTGTTGATATCGAAGATTCCCTTTACTAAATTAGTGAAGGAAGTCAC  
NDAI0C00590    ATACCAAAGATCAACAGAATTGCTAATATCAAAGATACCATTTACAAAATTAGTTAAAGAGGTGAC  
TBLA0G01890    ATACCAACGTTCCACGGAACTACTGATGTCGAAAATCCCGTTTGCCCGATTAGTCAAGGAAGTCAC  
TPHA0N01420    ATATCAAAGGTCGACAGATTTATTAATATCGAAAATTCCGTTTGCAAGATTAGTCAAGGAAGTCAC  
Kpol\_181.3     GTACCAGAGGTCAACTGAGTTGTTGATATCTAAAATACCATTTGCTAGGTTAGTGAAAGAGGTGAC  
ZYRO0F01144g   ATACCAGAGATCTACCGAATTGCTCATCTCAAAAATTCCATTTGCTAGATTGGTTAGGGAAGTTAC  
TDEL0B06850    ATACCAGCGATCGACCGAGCTGCTAATATCGAAGATCCCTTTCGCGAGGTTAGTCAAAGAGGTGAC  
KLLA0C12529g   ATATCAAAGATCCACAGATTTGCTTATATCCAGAATGCCCTTTGCGAGGCTAGTGAAAGAGGTAAC  
ABR083C        GTACCAGCGGTCGACGGAGCTGCTGATTTCGCGAATGCCGTTTGCGCGGCTGGTGAAGGAGGTGAC  
Ecym\_2307      GTATCAACGATCGACGGAGCTTCTAATTTCTCGAATGCCGTTTGCCAGATTGGTGAAGGAAGTTAC  
SAKL0B11198g   GTACCAACGAAGCACTGAGCTTTTGATATCGAAAATGCCATTTGCCCGTCTCGTAAAAGAGGTCAC  
KLTH0D08602g   GTATCAGCGAAGCACAGACCTTTTAATCTCAAAGATGCCCTTTGCACGCTTGGTCAAAGAGGTCAC  
Kwal\_26.8328   ATACCAAAGAAGCACGGATCTCCTGATATCTAAGATGCCGTTTGCGCGCCTAGTGAAGGAGGTAAC  
  
cons            \*\* \*\*        \* \*   \*  \* \*\*       \*\* \*\* \*\*  \*     \* \*\* \*  \*\* \*\*     
  
  
YKL049C        AGACGAGTTTACAACTAAAGATCAGGATTTACGTTGGCAGTCAATGGCGATTATGGCGTTACAGGA  
Smik\_11.199    GGATGAGTTCACGACTAAGGATCAAGATTTACGCTGGCAGTCAATGGCGATCATGGCACTACAAGA  
Skud\_11.180    AGACGAGTTTACAACTAAAGACCAAGATTTGCGCTGGCAATCGATGGCGATCATGGCGCTACAGGA  
Suva\_11.177    GGATGAGTTTACAACCAAAGATCAAGACCTGCGTTGGCAGTCAATGGCGATCATGGCGCTACAGGA  
CAGL0M13145g   AGAAGAATTTGCCGGTGAATCGCAAGATCTTCGTTGGCAATCAATGGCCATCCTCGCTCTTCAGGA  
KAFR0I01590    CGATGAGTTTACGTATCGAGACGAGAACCTTCACTGGCAATCTATGGCGATCGTAGCATTACAAGA  
KNAG0H00580    CAGTGACTTTGTATGGGAGAGCGAACCCCTAACTTGGCAGTCGATGGCGATCTTGGCATTGCAAGA  
NCAS0H03050    TGATGAATTTACCGTGGAAGACCAACAACTTCATTGGCAATCGATGGCCATTGTCGCGTTACAGGA  
NDAI0C00590    AGATCAATTTACCGTTGAGGAACAACAGTTACATTGGCAATCGATGGCAATTGTCGCGTTACAAGA  
TBLA0G01890    AGACGAGTTCACTCTAGACGAACAACAGTTCCGTTGGCAATCCATGGCCATTCTCGCATTGCAAGA  
TPHA0N01420    TGAACAATTCACCACAGAAGAACAAAATTTCAGATGGCAAAGCATGGCTATCCTGGCGTTACAAGA  
Kpol\_181.3     AGAGCAATTTACAACTGAGGAACAACAACTGCGGTGGCAGTCAATCGCGATCTTGGCCCTACAGGA  
ZYRO0F01144g   TGAACAATTTACCACGGAAGAACAACAGCCGCGATGGCAATCAATGGCGGTACTAGCTCTCCAAGA  
TDEL0B06850    CGATCAATTCACTACAGAGGAACAGCAATTGAGGTGGCAATCGATGGCTATTCTAGCCCTTCAGGA  
KLLA0C12529g   AGATCAATTCACTACCGAGAGCGAACCTCTTCGATGGCAATCCATGGCAATTATGGCTCTTCAAGA  
ABR083C        GGACCAGTTCACGACCGTCGACCAGCAGATGCGATGGCAGTCGATGGCCATCCTTGCGCTGCAAGA  
Ecym\_2307      GGATCAATTTACCACAGAAGAGCAACAGTTACGGTGGCAGTCGATGGCTATACTTGCGCTTCAAGA  
SAKL0B11198g   TGAACAATTTAGTACAGACGAACAGCAGCTCCGGTGGCAGTCTATGGCTATCCTAGCATTACAGGA  
KLTH0D08602g   AGACCAGTTCACCACCGAGGAACAGCAGTTGAGATGGCAGTCAATGGCTATAATGGCACTACAGGA  
Kwal\_26.8328   AGACCAATATACCACGGAGGAGCAACAATTAAGATGGCAGTCAATGGCAATAATGGCATTACAAGA  
  
cons                \* \*               \*          \*\*\*\*\*    \*\* \*\*  \*  \* \*\*  \* \*\* \*\*  
  
  
YKL049C        AGCAAGCGAAGCGTATCTGGTAGGATTATTGGAACATACAAACCTCTTGGCGCTGCATGCAAAAAG  
Smik\_11.199    AGCAAGTGAAGCCTATTTGGTGGGTCTATTGGAGCATACCAACCTTTTGGCATTGCACGCGAAAAG  
Skud\_11.180    GGCAAGTGAAGCATACTTGGTGGGGCTATTGGAGCATACCAACCTACTTGCGCTGCATGCGAAGAG  
Suva\_11.177    AGCAAGTGAAGCATATTTAGTGGGATTATTAGAGCATACCAACCTCTTAGCATTGCATGCGAAAAG  
CAGL0M13145g   AGCAAGTGAAGCTTACTTGGTAGGATTACTAGAGCATACAAATTTGTTGGCATTGCATGCTAAACG  
KAFR0I01590    GGCGAGCGAAGCTTACTTGGTGGGGCTATTGGAACATGCAAATTTACTAGCCCTACATGCTAAAAG  
KNAG0H00580    AGCAAGCGAGGCTTATCTGGTGGGCCTCTTGGAACATGCCAATCTACTGGCTTTGCACGCGAAAAG  
NCAS0H03050    AGCCAGTGAAGCTTATTTAGTGGGTTTATTGGAGCATGCCAATTTATTAGCCATACATGCCAAGAG  
NDAI0C00590    GGCTAGTGAAGCCTACTTGGTTGGCCTTCTAGAACATGCAAATCTACTTGCATTACATGCCAAGAG  
TBLA0G01890    GGCCAGTGAAGCATATCTGGTGGGTCTACTAGACCACACCAACTTGTTGGCACTGCATGCAAGACG  
TPHA0N01420    AGCAAGTGAAGCGTACCTGGTAGGGCTACTAGAACACACAAACCTTCTAGCGTTGCATGCAAGAAG  
Kpol\_181.3     GGCCAGCGAGGCCTATCTTGTTGGATTGTTAGAACACACGAACTTGCTGGCACTTCATGCTAAACG  
ZYRO0F01144g   AGCCAGTGAGGCCTATTTAGTGGGATTGCTAGAACATACGAATTTACTAGCACTTCATGCAAAAAG  
TDEL0B06850    GGCTAGTGAAGCGTACCTTGTTGGGCTACTAGAGCATACTAATCTGCTCGCGTTGCATGCCAAAAG  
KLLA0C12529g   AGCAAGTGAAGCCTACTTAGTCGGTTTATTAGAGCATACAAATCTATTGGCACTTCATGCTAAAAG  
ABR083C        GGCGAGCGAAGCGTACATCGTGGGTCTCCTGGAACACACGAACCTGCTGGCGCTGCATGCGAAGCG  
Ecym\_2307      AGCAAGTGAAGCATATCTAGTTGGTCTTTTAGAGCACACAAATCTGCTTGCGTTACATGCAAAAAG  
SAKL0B11198g   GGCTAGTGAAGCGTACTTGGTTGGTCTACTGGAGCACACAAACCTGTTAGCCTTACACGCTAAAAG  
KLTH0D08602g   AGCAAGTGAAGCATACTTAGTTGGTCTCTTGGAACACACAAACCTATTAGCGTTACATGCAAAGAG  
Kwal\_26.8328   AGCGAGCGAAGCATATTTAGTGGGACTTTTAGAGCATACAAACTTATTGGCGCTGCACGCCAAACG  
  
cons            \*\* \*\* \*\* \*\* \*\*  \* \*\* \*\*  \*  \* \*\* \*\*  \* \*\*  \*  \* \*\*  \* \*\* \*\* \*   \*  
  
  
YKL049C        AATTACTATAATGAAGAAAGACATGCAACTAGCAAGAAGAATCAGGGGACAGTTTATTTAG  
Smik\_11.199    GATAACTATAATGAAGAAGGACATGCAACTAGCAAGGAGAATCAGAGGACAATTCATTTAA  
Skud\_11.180    AATAACCATAATGAAGAAGGATATGCAACTAGCAAGGAGAATTAGAGGACAGTTCATTTAA  
Suva\_11.177    GATAACCATAATGAAAAAAGACATGCAACTGGCAAGGAGAATTAGAGGACAATTCATTTAG  
CAGL0M13145g   TATCACGATTATGAAGAAGGACATGCAATTAGCTAGGAGAATAAGAGGACAGTTCATCTGA  
KAFR0I01590    GGTTACACTCATGAAAAAAGACGTACAACTGGCACGAAGAATCAGAGGTCAATTTATATAG  
KNAG0H00580    AGTCACAGTGACCAAAAAGGACATACAATTGGCTAGACGGATCAGAGGTCAGTTTATCTAG  
NCAS0H03050    GATCACATTAATGAAGAAGGATATACAGTTGGCCAGAAGAATTAGAGGTCAATTCATTTAG  
NDAI0C00590    GATCACTCTAATGAGGAAAGACATACAATTAGCAAGAAGAATCAGAGGGCAATTCATTTGA  
TBLA0G01890    TATTACCATAATGAAAAAAGATATGCAATTGGCAAGAAGAATTAGAGGTCAATTTATTTAA  
TPHA0N01420    AATAACAGTAATGAGAAAAGACATGCAACTGGCAAGAAGAATTAGAGGTCAATTTATATAG  
Kpol\_181.3     CATAACTGTCATGCGCAAGGATATGCAATTGGCAAGAAGAATCAGAGGACAATTCATTTAA  
ZYRO0F01144g   AATTACAATCATGAGGAAAGATATGCAGCTAGCGAGAAGAATAAGAGGGCAGTTTCTTTGA  
TDEL0B06850    AATTACGATAATGAGGAAAGATATGCAACTGGCGAGAAGGATACGAGGCCAATTTTTGTAG  
KLLA0C12529g   AATTACAATTATGAGAAAGGACATGCAACTAGCTAGAAGGATACGAGGTCAATTTATTTGA  
ABR083C        TGTGACGGTGATGCGGAAGGATATGCAGCTAGCGCGGCGGATCCGCGGCCAGTTTATATAG  
Ecym\_2307      GATCACGATCATGCGCAAGGACATGCAATTGGCAAGGAGGATACGGGGTCAGTTTATTTAA  
SAKL0B11198g   AATTACCATTATGAAGAAAGATATGCAGCTGGCAAGGAGAATTAGGGGCCAGTTTATTTGA  
KLTH0D08602g   AATCACTATTATGAGGAAAGACATGCAATTGGCCAGGAGAATTCGAGGCCAGTTTATTTAG  
Kwal\_26.8328   AGTAACGATAATGAGGAAAGACATGCAATTGGCCAGAAGAATTCGCGGCCAATTTATCTAG  
  
cons             \* \*\*  \* \*     \*\* \*\*  \* \*\*  \* \*\*  \*  \* \*\*  \* \*\* \*\* \*\*  \* \*    
  
  
  
  
  
