# Supplementary figures and images for: Loss of inner kinetochore genes is associated with the transition to an unconventional point centromere in budding yeast
Source: PeerJ. 2020 Sep 29;8:e10085. doi: 10.7717/peerj.10085 (PMC7531349; doi:10.7717/peerj.10085)

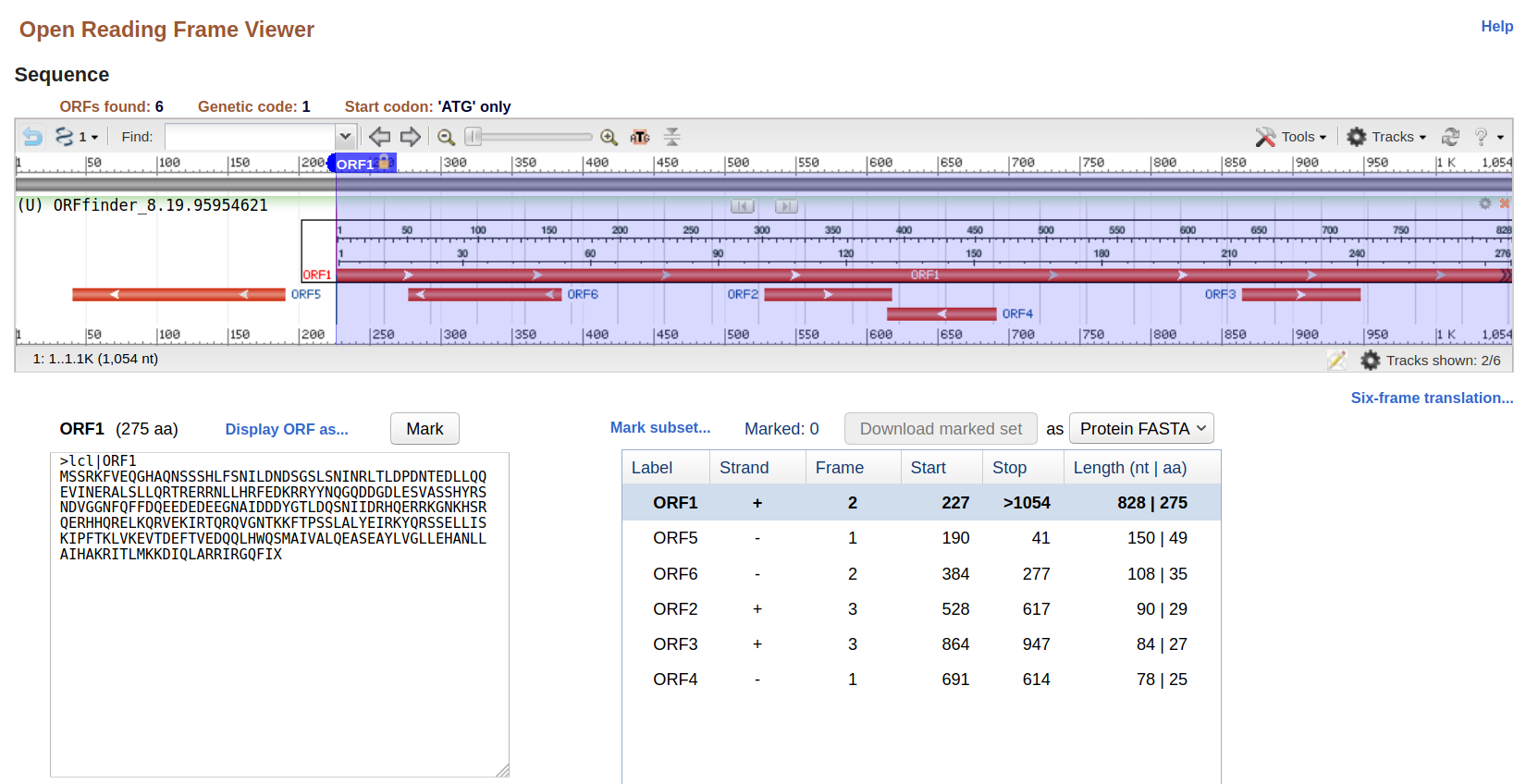

Supplement: Supplemental Information 4 — To rule out the possibility of erroneous annotation of shorter ORF’s in non-naumovozyma species I extracted the genomic sequence found between genes flanking CENPA (CENH3 and CSE4 homolog) and searched them for ORF’s using the NCBI ORF finder program with default settings. Screenshots of the results of the ORF finder program are also provided. [file peerj-08-10085-s004.zip › Supplementary_Material_S4/NCAS_ORFS_CSE4.png]

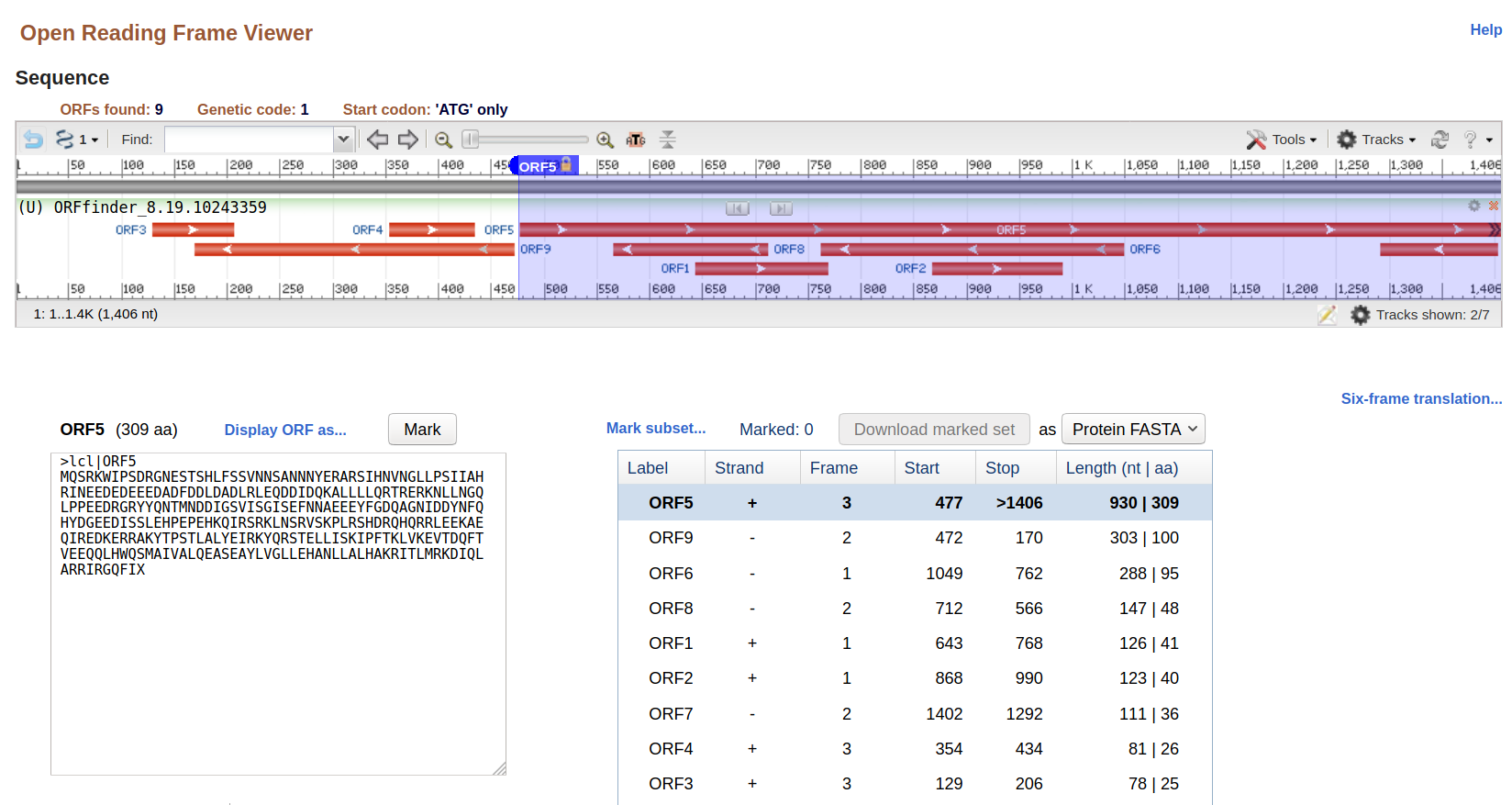

Supplement: Supplemental Information 4 — To rule out the possibility of erroneous annotation of shorter ORF’s in non-naumovozyma species I extracted the genomic sequence found between genes flanking CENPA (CENH3 and CSE4 homolog) and searched them for ORF’s using the NCBI ORF finder program with default settings. Screenshots of the results of the ORF finder program are also provided. [file peerj-08-10085-s004.zip › Supplementary_Material_S4/NDAI_ORFS_CSE4.png]
